# Acoustic Radiation Effects On Bone Conduction Threshold Measurement

**DOI:** 10.1590/S1808-86942010000500020

**Published:** 2015-10-22

**Authors:** Renata das Merces Bastos de Matos, Silvania de Paula Valle, Anna Marcella Neves Dias, Teresa Maria Momensohn dos Santos, Isabel Cristina Gonçalves Leite

**Affiliations:** 1 Audiologist; 2 Speech and hearing therapist - UNIPAC-JF; 3 MSc in Speech and Hearing Therapy - Pontifícia Universidade Católica de São Paulo - PUCSP, Professor of the Biomedicine and Veterinary Medicine Programs; Audiologist - Clínica de Otorrinolaringologia de Juiz de Fora; 4 PhD in Human Communication Disorders - UNIFESP/SP; Full Professor - Department of Speech and Hearing - Therapy -PUCSP. Head of the IEAA; 5 PhD in Public Health; Adjunct Professor - Medical School -UFJF

**Keywords:** radiation, audiometry, pure-tone, bone conduction

## Abstract

**Abstract:**

Acoustic radiation is the sound energy escape from a bone vibrator that may be detected by air conduction mechanisms. The presence of acoustic radiation may result in an unreal bone conduction threshold, promoting an unreal air/bone gap in the high frequencies.

**Aim:**

aim to analyze the acoustic radiation effect on the extension of air/bone gap at 2,000, 3,000 and 4,000 Hz.

**Materials and Method:**

our clinical and experimental study had a sample of 101 individuals, who matched inclusion criteria: to have an air/bone gap higher than 10 dB in the frequencies of 2,000; 3,000 and 4,000 Hz. All of them had their bone conduction threshold measured in two conditions: open ear canal and closed ear canal.

**Results:**

we found that major differences between the two conditions evaluated occurred at the 4,000 Hz; data analysis showed significant difference in the extension for the air/bone gap; analysis of the number of cases of mixed hearing loss that changed to sensorineural was significant too.

**Conclusion:**

These studies concluded that when the MAE is occluded, the acoustic radiation phenomenon is controlled or avoided, enabling bone measures at the frequencies of 3,000 and 4,000Hz to be more accurate.

## INTRODUCTION

Speech and hearing therapists are professionals who study hearing, its mechanisms, the way through which the auditory system processes sounds and also the procedures used to evaluate its disorders. Audiometric tests, or threshold tonal audiometry, are considered the most basic test to assess hearing. Based on the measures of hearing thresholds obtained from air and bone conductions, it is possible to establish the first topodiagnosis in audiology. Therefore, the examiner must have full control over artifacts or errors which can compromise the accuracy and reliability of these measures. The examiner is responsible for knowing the strategies which will help him/her obtain such results.

The literature on bone conduction measures presents studies associated with: the technical limits of the bone vibrator; the pressure and placement of the vibrator on the mastoid; use of masking; the influence of the middle ear diseases and the phenomena of occlusion and acoustic radiation. Practice in clinical audiology leads us to question to what extent the results from the bone conduction assessment of an individual is true or if it is the product of interference of actions or facts which occur during audiometry.^1^

One clinical concern regarding bone conduction audiometry is the effect of radiation on the acoustic energy emitted by the bone vibrator to the external ear canal. This signal, now transmitted through air conduction, can be sufficiently intense in order to serve as an additional clue to patients when they answer to stimuli presented on the bone pathway. The bone thresholds of this patient can be corrupted, appearing to be better than they really are. Such finding would produce invalid air-bone gaps, especially in the higher frequencies. The interpretation of this audiometric test can also be compromised.^2^

The possibility of hearing the acoustic stimuli irradiated by the bone vibrator, also through air conduction, because of the transmission of its energy to inside the external ear canal has been discusses by many authors.^3^, ^4^, ^5^, ^6^, ^7^

A normal-hearing individual hears through the acoustic radiation of a bone vibrator at a level which is subjectively more intense than the energy produced by the vibrator. When this phenomenon happens, the bone conduction measure will seem better than expected and a false acoustic gap will be recorded. The size of this gap will correspond to the amount of acoustic radiation present.^5^

A study measured the acoustic radiation at different frequencies and with different bone vibrators (Radioear B-71, B-72, and B-70A BC) in an artificial mastoid. Bell et al. (1980), Shipton et al. (1980), and Frank & Holmes (1981) measured the acoustic radiation at the entry point of the human external ear canal (EEC) using RadioEar bone vibrators placed on the mastoid. All these studies showed that the maximum acoustic radiation happened at 4000 Hz, especially when using RadioEar, B-71 and B-72 bone vibrators.^8^,^9^

The sound pressure level (SPL) was measured on the EEC for the frequencies of 2000 and 4000 Hz, in 50 individuals. The bone vibrator (RadioEar B-71) was positioned on the mastoid and on the forehead. A probe microphone was placed on the EEC, contralateral to the stimulated mastoid. They found clinically significant air-bone gaps (higher than 10dB) because of the acoustic radiation and it was clearer on the 4,000 Hz frequency.^2^

In a recent study the authors reported the influence of the ear plug on bone threshold measures at 2,000; 3,000 and 4,000 Hz with the RADIOEAR B-71 bone vibrator. The bone threshold was established on 36 ears with the EEC open and EEC occluded (ear plug on). This study proved the phenomenon of the acoustic radiation on the frequencies of 2, 3 and 4 kHz when the bone conduction was evaluated, especially at 3 kHz, in 70% of the cases.^10^

The acoustic radiation effect was investigated in 148 individuals with mixed hearing loss (mean age of 44.7 years) by means of air and bone tonal audiometry. The bone conduction thresholds for the frequencies of 2,000; 3,000 and 4,000 Hz, were obtained in two different situations: open EEC and closed EEC (the patient occluded the ear canal with his own index finger). The authors observed that on the latter situation, the air/bone gap changed in many of the individuals, which altered the interpretation of the results from the mixed to the sensorineural type.^11^

As we find an air/bone gap higher than 10 dB, in patients with a history of inner ear disease, it is important to redo the measure occluding the EEC. Working with occupational health, the authors have found many audiometric exams with air/bone gaps higher than 10 dB on the frequencies of 3,000 and 4,000 Hz. How can one explain such gap in a presentation which cause usually generates a sensorineural hearing loss? How can we interpret such result?^12^

The goal of the present study was to assess the effects of this acoustic radiation on the air/bone gap magnitude on the frequencies of 2,000; 3,000 and 4,000 Hz.

## MATERIALS AND METHODS

This study was exploratory, descriptive, and aimed at checking the interference of acoustic radiation on the bone conduction tonal threshold audiometry in the frequencies of 2,000; 3,000 and 4,000 Hz.

Data collection was carried out in a clinic in Juiz de Fora - MG, from January through February of 2009, after approval from the Ethics in Research Committee (SISNEP # 112/ 08). All the patients signed the Informed Consent Form (Attachment A) in order to participate in the study.

The sample was made up of 101 individuals, 11 ears from females and 130 ears from males, with age ranging between 20 and 70 years (mean of 44.7 years, SD 12.3 years). We selected those participants who met the following inclusion criteria: no external ear canal obstruction or foreign body and air/bone gap higher than 10 dB on the frequencies of 2,000; 3,000 and/or 4,000 Hz. We took off the study those individuals who did not have air conduction response on the frequencies of 2,000; 3,000 and 4,000 Hz and normal ears.

All the participants were submitted to: interview, external ear canal inspection, air conduction threshold tonal audiometry with supra-aural phones and bone conduction threshold tonal audiometry with the RadioEar B-71 bone vibrator. We used the AMPLAID A137 clinical audiometer calibrated according to the ISO R 389/ANSI S 3.6-1996/ ISO 7566/ ANSI S 3.43-1992 standards. All the tests were carried out in a sound-treated booth.

All the participants who had a gap between the air and bone conduction thresholds in the frequencies of 2,000; 3,000 and 4,000 Hz, had the bone conduction thresholds reassessed with the occlusion of the external ear canal under test with the patient's own index finger.

The data collected were stored in the Microsoft Excel 2007®software, in a spreadsheet with: gender, age, air conduction threshold for the following frequencies: 250; 500; 1,000; 2,000; 3,000; 4,000; 6,000; 8,000 Hz from both ears; bone conduction threshold in the frequencies of 500; 1,000; 2,000; 3,000; 4,000 Hz from both ears with the EEC open and bone conduction threshold for the frequencies of 2,000; 3,000; 4,000 Hz for both ears with the EEC occluded.

## STATISTICAL METHOD

The Data collected were submitted to a descriptive statistical analysis, with values for the mean, standard deviation, median, minimum and maximum and also the analysis of the differences between the two conditions evaluated - open EEC and closed EEC, in order to establish the level of significance. In order to compare the frequencies between the groups we used the chi-square (c2) with a 5% significance level.

We used the SPSS 8.0 software.

## RESULTS

Table 1 depicts the descriptive analysis of the gap values found in the two situations (open external ear canal x closed external ear canal), for the frequencies evaluated (2,000; 3,000 and 4,000 Hz).

**Table 1:** Descriptive analysis of the air/bone gap values (dB) found in the population studied (n=141 ears).

		Mean dB	SD dB	Median dB
2000 Hz	Open EEC	13,4	12,7	10
	Closed EEC	13,7	12,9	10
3000 Hz	Open EEC	23	14,6	25
	Closed EEC	24,8	14,9	25
4000 Hz	Open EEC	26,4	13,5	25
	Closed EEC	33,3	14,9	35

Legend: EEC - External Ear Canal

In Charts 1, 2 and 3 it is possible to see the results from the crossed plotting between the number of ears in which air/bone gap changed between the two situations: open EEC and closed EEC.

Chart 1. We notice that in 2,000 Hz there were no changes in the air-bone gap values of the ears investigated.

**Chart 1:** Distribution of the data obtained from the analysis of air/bone gap value changes on the Open EEC X Closed EEC situations for this sample of 141 ears, in the frequency of 2,000 Hz.

					GAP 2,000 Hz Closed EEC				
dB HL	0	5	10	15	20	25	30	40	Total
	0	103							103
	5	2	13						15
	10		1	5					6
GAP2,000	15				3				3
Hz Open	20					6			6
EEC	25						4		4
	30						1	1	2
	40						1	1	2
Total	105	14	5	3	6	5	2	1	141

Chart 2. In the frequency of 3,000 Hz it was possible to notice that with the change in the air-bone gap values, 7 ears which tests had been interpreted as mixed type became sensorineural hearing loss.

**Chart 2:** Distribution of the data obtained from the analysis of the air/bone gap value changes in the Open External Ear Canal x Closed External Ear Canal, for a sample of 141 ears, in the frequency of 3,000 Hz.

GAP 3,000 Hz Closed EEC													Total
	gap CA/CO dB HL	0	5	10	15	20	25	30	35	40	45	55	
	0	27											27
	5	14	14										28
	10	6	9	18									33
	15	4		3	9								16
	20				1	11							12
GAP3000 Hz	25						9						9
Open EEC	30							1					1
	35									5			5
	40									8			8
	50										1		1
	55											1	1
	Total			51	23	21	10	11	9	1	581	1	141

Chart 3. In the 4000 Hz frequency, the number of ears changing classification as to the type of hearing loss was 45, in other words, in 45 individuals the audiometric test had been interpreted as mixed, when, in fact these individuals had sensorineural hearing loss.

**Chart 3:** Distribution of the data obtained from the air/bone gap value changes analysis in the Open External Ear Canal X Closed External Ear Canal, for the sample of 141 ears, in the frequency of 4,000 Hz.

GAP 4,000 Hz Closed EEC											
		0	5	10	15	20	25	30	35	40	Total
	0	3									3
	5	67									13
	10	17	7	5							29
	15	16	5	4	5						30
GAP 4,000Hz	20	8	6	3	8	10					35
Open EEC	25		1	2	1	2	4				10
	30						2	7			9
	35						11	3			5
	40								1	5	6
	45								1		1
	Total	50	26	14	14	12	7	8	5	5	141

We found a statistically significant difference in the frequency of status change (mixed x sensorineural) for the acoustic radiation phenomenon (p<0.001), nonetheless there was no age-wise difference (<45 or >=45 years) for the right ear (p=0.61) as well as for the left ear (p=0.47).

We also noticed a statistically significant difference between the presence and the absence of differences between the closed and open bone conduction and the frequencies in which the acoustic radiation happened (p<0.001).

## DISCUSSION

As to the comparison done through the bone conduction of the open and closed EEC at 2,000 Hz, there was no statistically significant difference (Table 1). This result differed from those by Cili (2008) and from those by Momensohn-Santos et al. (2008), who found differences, showing that there was a worsening in the bone conduction threshold. However, there was a statistically significant difference in the frequencies of 3,000 and 4,000 Hz, as it happened in the studies led by Cili (2008), Momensohn Santos et al. (2008). Such findings confirmed the presence of acoustic radiation in the high frequencies as suggested by Dirks and Malmquist, (1969); Tonndorf (1972); Lightfoot (1979), Bell et al. (1980), Shipton et al. (1980), Frank & Holmes (1981) and Silman & Silverman (1991).

The findings presented on Charts 1, 2 and 3 show that, with the occlusion of the EEC, it is possible to avoid the acoustic radiation phenomenon, since there was a worsening in the bone threshold when the EEC was occluded. These findings prove that the energy escape from the bone vibrator could not reach the cochlea because the EEC was blocked, as suggested by Silman & Silverman (1997).

With such procedure, it was possible to identify numerous exams which could have been interpreted as mixed, and were actually, sensorineural.

Silman & Silverman (1997) commented that among the factors which produced artifacts on the bone vibration results is the effect of acoustic radiation.

## CONCLUSION

The Findings from this study proved that when the EEC is occluded, the acoustic radiation phenomenon is controlled or avoided, enabling more accurate bone conduction measures in the frequencies of 3,000 and 4,000 Hz.
